# Poor Prognostic Biomarker KIAA1522 Is Associated with Immune Infiltrates in Hepatocellular Carcinoma

**DOI:** 10.1155/2023/3538928

**Published:** 2023-01-31

**Authors:** Yongjie Shi, Qiwen Xiao, Sicong Huang, Qimou Pan, Hongyun Jia, Qiang Zhou, Jie Wei, Jiale Kang, Zhe Li, Yingyu Hu

**Affiliations:** ^1^Department of Clinical Laboratory, The Second Affiliated Hospital of Guangzhou Medical University, Guangzhou, Guangdong, China; ^2^The Second Affiliated Hospital of Guangzhou University of Chinese Medicine, Guangzhou, Guangdong, China; ^3^Department of Neurology, The Second Affiliated Hospital of Guangzhou University of Chinese Medicine, Guangzhou, Guangdong, China; ^4^Department of Hospital Management, Southern Medical University, Guangzhou, Guangdong, China

## Abstract

**Background:**

The prognosis is poor for hepatocellular carcinoma (HCC), a tumor and cancer associated with inflammation that is common. New data showed that significant levels of KIAA1522 were expressed in HCC tissues and cell lines, suggesting that KIAA1522 may be a highly useful prognostic marker for HCC. However, its biochemical processes and impacts on the immune system go deeper.

**Objective:**

To verify the significance of KIAA1522 in HCC and investigate its related carcinogenic mechanisms.

**Methods:**

Studies examining the relationship between KIAA1522 expression and clinical-pathologic characteristics in HCC have been checked in the Cancer Genome Atlas (TCGA) database. A receiver operating characteristic (ROC) curve was used to assess the diagnostic efficacy of KIAA1522 in HCC. Western blot analysis was used to find the presence of the KIAA1522 protein in the tumor and paraneoplastic tissues of eight randomly chosen HCC patients. The GSVA program in R language was used to evaluate the relationship between KIAA1522 and immune cell infiltration in HCC. We searched the Search Tool for the Retrieval of Interacting Genes (STRING) database for interacting proteins connected to the expression of KIAA1522. Pathways were involved in the enrichment analysis of KIAA1522 to anticipate potential mechanisms through which KIAA1522 may affect immunological infiltration.

**Results:**

Our study found that KIAA1522 was commonly elevated in HCC tumor tissues and that it also signaled a bad outcome. We found an inverse link between KIAA1522 and cytotoxic cells and an inverse relationship between KIAA1522 and Th2 cell infiltration. In STRING analysis, the top 5 coexpressed proteins of KIAA1522 were BAIAP2, NCK2, TSNAXIP1, POGK, and KLHL31. The effect of KIAA1522 on HCC may entail cell cycle alteration, an immunological response, and suppression of the PPAR signaling pathway.

**Conclusion:**

High expression of KIAA1522 was linked to HCC immune cell infiltration, disease progression, and a poor prognosis.

## 1. Introduction

According to data on cancer worldwide, liver cancer accounts for 782,000 fatalities and 841,000 new cases annually [1]. Among all cancers, it has the fourth-highest incidence and sixth highest fatality rates [2–5]. Hepatocellular carcinoma (HCC) makes up between 75 and 85 percent of primary liver cancers [4], and more than half of HCC cases are found in China [6]. Since the majority of HCC patients receive their diagnoses at an advanced stage, there are not many antitumor medications or viable therapy options. It is not encouraging that 40% of HCC patients pass away within 5 years [7, 8]. Therefore, finding new markers for the diagnosis and treatment of HCC has always been a hot topic in academia [9].

When present in the tumor microenvironment (TME), the degree of immune cell infiltration plays a crucial role in cancer start, development, metastasis, and treatment resistance [10, 11]. It is reported that the subsets of immune cells such as tumor-associated macrophages (TAMs), myeloid-derived suppressor cells (MDSCs), tumor-associated neutrophils (TANs), regulatory T cells (Tregs), cancer-associated fibroblasts (CAFs), CD8+ cytotoxic T lymphocytes (CTLs), and tumor-infiltrating lymphocytes (TILs) are critical immunosuppressive components in the TME of HCC, which could promote HCC growth and invasion [12].

It is important to investigate the role of the big protein-coding gene KIAA1522. The mRNA expression of KIAA1522 was elevated in a number of malignancies, according to data from the Gene Expression Atlas and The European Bioinformatics Institute databases, which showed that abnormal KIAA1522 expression may have a role in the pathogenesis and growth of cancer [13]. Recent research demonstrated that elevated KIAA1522 expression accelerated the evolution of HCC by activating the Wnt/*β*-catenin pathway and predicted a poor prognosis in HCC patients [14, 15]. The function of KIAA1522 in human cancer immunology, however, has hardly ever been thoroughly examined. Then, we speculate that the expression of KIAA1522 may be related to immunological subtypes, developing immune biomarkers, and TILs analysis in HCC.

We conducted a comprehensive analysis of the value of KIAA1522 in HCC prognosis and tumor immune infiltration using RNA sequencing data retrieved from the TCGA database, along with receiver operating characteristic (ROC) curve, differentially expressed gene (DEG) analysis, gene ontology (GO) term analysis, Kyoto Encyclopedia of Genes and Genomes (KEGG) pathway analysis, single-sample gene set enrichment analysis (ssGSEA), gene set enrichment analysis (GSEA), Kaplan–Meier survival analysis, and logistic and Cox regression analysis. This study's goal was to investigate the potential of KIAA1522 as an anticancer immunotherapy in human cancer in an effort to shed light on a cutting-edge strategy for HCC anticancer treatment.

## 2. Materials and Methods

### 2.1. Data Acquisition and Preprocessing

The TIMER2.0 database [16] (https://timer.comp-genomics.org/) was used to examine the amounts of KIAA1522 mRNA in different tumor types. The importance of KIAA1522 in HCC was confirmed through a clinical investigation and examination of the TCGA database [17] (https://portal.gdc.cancer.gov/) and UALCAN database [18] (https://ualcan.path.uab.edu/analysis.html). The clinical features and gene expressions of 374 HCC patients and 50 normal tissues' RNA-Seq data, which were converted from Fragments Per Kilobase Million (FPKM) to Transcripts per Million (TPM) format while retaining the original clinical data, were analyzed. By using the pROC program in R to create ROC curves, the diagnostic effectiveness of KIAA1522 for HCC was assessed [19]. To examine the variation in KIAA1522 mRNA expression across HCC, normal samples, and paracancer samples, we used paired tests. The correlation with clinical information, such as patient survival time, pathological stage, and clinical stage, was examined. In order to evaluate the predictive value of KIAA1522, K-M curves [20] were plotted.

### 2.2. Western Blot

Centrifuging the protein-containing lysate was followed by a western blot analysis. Cell lysates or immune precipitates were resolved using SDS-PAGE (Thermo Fisher Scientific, Shanghai, China), and proteins were then transferred to polyvinylidene difluoride membranes (Roche, Basel, Switzerland). The membranes were then kept in 5% skimmed milk blocking for 1 hour, treated with secondary HRP antibody (Thermo Fisher Scientific, Shanghai, China) for 2 hours at room temperature, and then incubated with anti-KIAA1522 antibody (Abcam, Cambridge, MA, USA) overnight. Software called Image J 1.35 V was used to display the protein bands. Eight pairs of HCC tumor tissues that had been histopathologically and clinically diagnosed at the Second Affiliated Hospital of Guangzhou Medical University—along with corresponding normal tissues—were used in this study. The institutional research ethics committee's approval and prior patient consent were acquired. Each sample was exclusively utilized for the study.

### 2.3. Differentially Expressed Gene Analysis

For our analysis, we used the DESeq2 R package's unpaired *t*-test, adjusted for *P* < 0.05 and |Log2FC| ≥ 1.5 [21], and we classified the expression data into two groups with reference to the median KIAA1522 expression level.

### 2.4. Immune Infiltration Analysis

Utilizing the single-sample gene set enrichment analysis (ssGSEA) technique by the GSVA package, it was possible to assess the abundance of various immune cells among KIAA1522 high expression groups and low expression groups in HCC using the TCGA data and the expression levels of genes from the published list of signature genes [22]. *P* < 0.05 was regarded as statistically significant. The immunological correlation score analysis and immune checkpoint analyses were plotted using the ggplot2 R package [23]. Immune cell infiltration between groups with high and low expression levels of KIAA1522 was analyzed using Spearman correlation and the Wilcoxon rank sum test.

### 2.5. Protein-Protein Interaction Network

We examined the PPI network of the functional interactions between proteins, which was available on the STRING website (https://string-db.org/) [24] and displayed by Cytoscape software [25]. An interaction score ≥0.4 was necessary for these genes. A maximum number of interactors = 0, and the genes were imported into Cytoscape to screen with a degree cut-off = 2, haircut on, node score cutoff = 0.2, *k* core = 2, and max. depth = 100.

### 2.6. Enrichment Analysis

For enrichment analysis, we used the Kyoto Encyclopedia of Genes and Genomes (KEGG) and Gene Ontology (GO) from the ClusterProfiler R package [26]. Results were deemed significantly enriched if the false discovery rate (FDR) <0.25 and the adjusted *P*-value <0.05.

### 2.7. Statistical Analysis

In this study, online tools were used for statistical analysis and graphical visualization. The test level *α* = 0.05. *P* < 0.05 was defined as statistically significant.

## 3. Results

### 3.1. Differential Expression Analysis of KIAA1522 in HCC

**Figure 1:**
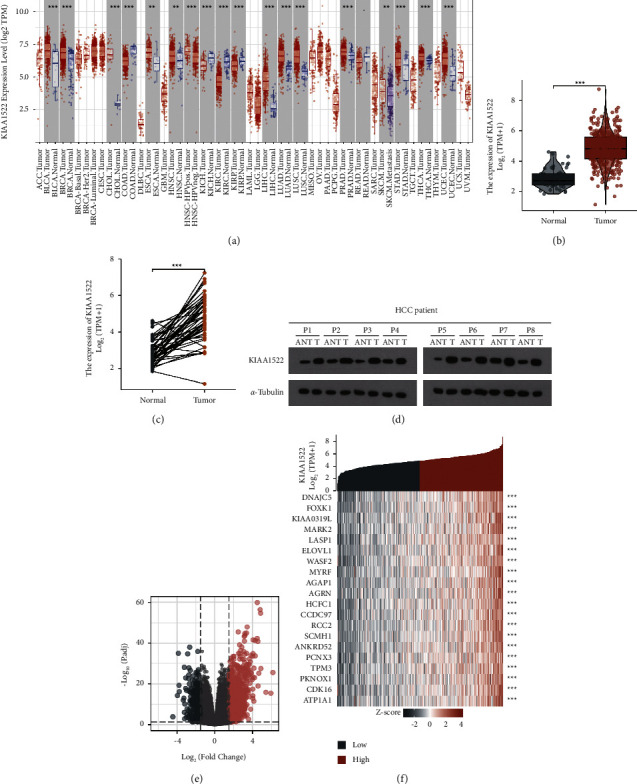
The KIAA1522 of differentially expressed genes (DEGs). (a) Differential expression levels of KIAA1522 in different tumor and paraneoplastic tissues in TIMER2.0 database. (b, c) The expression difference of KIAA1522 between HCC and normal control. (d) KIAA1522 protein differentially expressed in 8 randomly selected HCC patients' paired tissue. (e) The volcano plot of KIAA1522 differentially expressed RNAs. (f) 20 genes correlated to KIAA1522 of the heat map; ^*∗*^*P* < 0.05, ^*∗∗*^*P* < 0.001, and ^*∗∗∗*^*P* < 0.01.

The increased KIAA1522 mRNA levels were observed in various tumors, including bladder urothelial carcinoma, breast invasive carcinoma, cholangiocarcinoma, colon adenocarcinoma, esophageal carcinoma, head and neck squamous cell carcinoma, kidney chromophobe, kidney renal clear cell carcinoma, kidney renal papillary cell carcinoma, liver hepatocellular carcinoma, lung adenocarcinoma, lung squamous cell carcinoma, pancreatic adenocarcinoma, rectum adenocarcinoma, skin cutaneous melanoma, stomach adenocarcinoma, thyroid carcinoma, and uterine corpus endometrial carcinoma (Figure 1(a)), suggesting an essential role in tumorigenesis and development. In addition, KIAA1522 was markedly upregulated in HCC (Figures 1(b) and 1(c)). KIAA1522 protein levels were shown to be higher in 8 pairs of randomly chosen HCC tissue by Western blot (Figure 1(d)). In the volcano map, the red dots represent a total of 975 genes whose expression was considerably upregulated, and the blue dots represent a total of 361 genes whose expression was significantly downregulated. The figures demonstrate that among the numerous identified genes, 1336 differentially expressed genes were produced (Figure 1(e)). Twenty genes with differential expression were used to create a heat map of the clustering of KIAA1522 (Figure 1(f)).

### 3.2. Associations between KIAA1522 Expression and Clinical Characteristics

253 male and 121 female HCC patients in total were enrolled in this study.  Table 1 contains clinical data that were examined in relation to KIAA1522. KIAA1522 was statistically different from overall survival (OS) events by Fisher's exact test (*P*=0.039). The Chi-square test result revealed that KIAA1522 expressions were statistically significant, respectively, in the T stage (*P*=0.031) and histological grade (*P* < 0.001). And the trends of correlation with the pathologic stage (*P*=0.070) and vascular invasion (*P*=0.014) were found. Furthermore, the Wilcoxon rank-sum test revealed a significant correlation between KIAA1522 and alpha-fetoprotein (AFP) (ng/ml) (*P* < 0.001) and body mass index (BMI) (*P* < 0.001). However, gender (*P* < 0.047) and age (*P*=0.020) showed statistically significant variations. Other clinicopathological characteristics were not statistically linked with KIAA1522.

**Figure 2:**
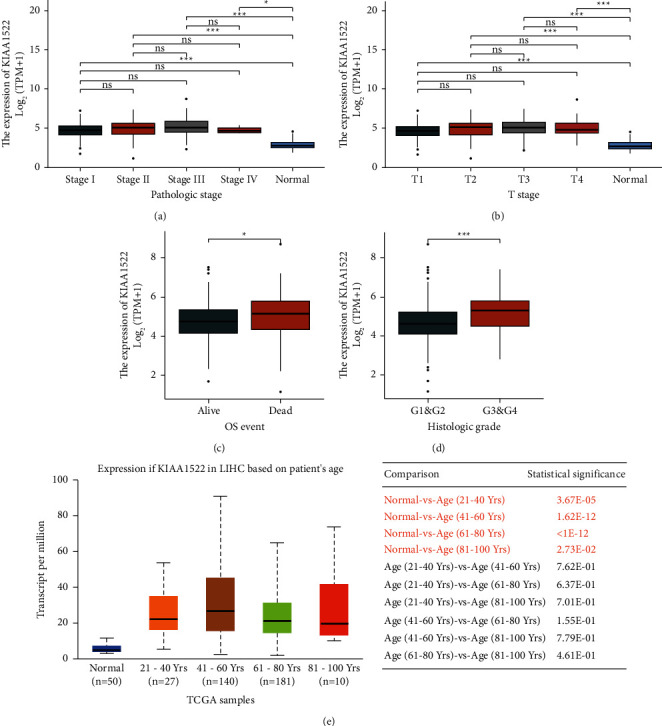
Association between the KIAA1522 expression and different clinicopathologic characteristics. (a) Association between the KIAA1522 expression and the pathologic stage of HCC. (b) T stage. (c) OS event. (d) Histologic grade. (e) KIAA1522 expression at different ages.

Welch's one-way ANOVA proved that KIAA1522 was correlated with the T stage (Figure 2(a)) and pathologic stage (Figure 2(b)), which had significant differences in the OS event (Figure 2(c)) and histologic grade (Figure 2(d)) concurrently. As well as, there was no significant difference in KIAA1522 expression among various age groups by *t*-test analysis in the UALCAN database (Figure 2(e)).

Logistic regression analysis showed that KIAA1522 was significantly correlated with the T stage (*P*=0.003), histologic grade (*P* < 0.001), AFP (*P* < 0.001), tumor status (*P*=0.022),and had a trend of correlation with pathologic stage (*P*=0.088) and vascular invasion (*P*=0.082) (Table 2). In the Cox regression model, univariate Cox regression indicates that the T stage (*P* < 0.001), M stage (*P*=0.017), KIAA1522 (*P* < 0.001), and pathologic stage (*P* < 0.001) were positively correlated with poor prognosis of HCC (Table 3).

**Figure 3:**
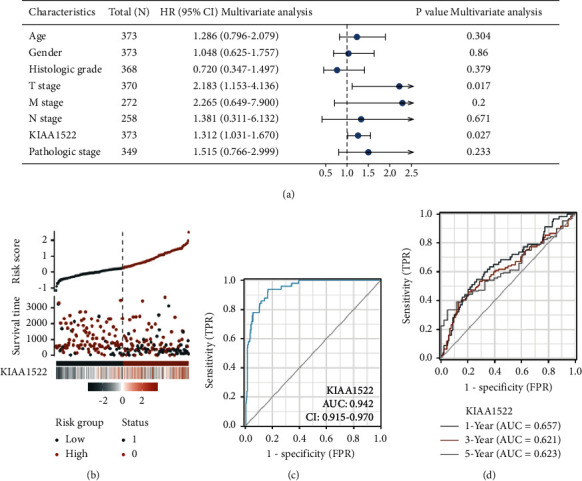
The prognostic value of KIAA1522 in HCC. (a) Multivariate Cox regression visualized in the forest plot. (b) KIAA1522 expression distribution and survival status, 0: dead; 1: alive. (c) Diagnostic ROC curve of KIAA1522. (d) Time-dependent ROC curve of KIAA1522.

**Figure 4:**
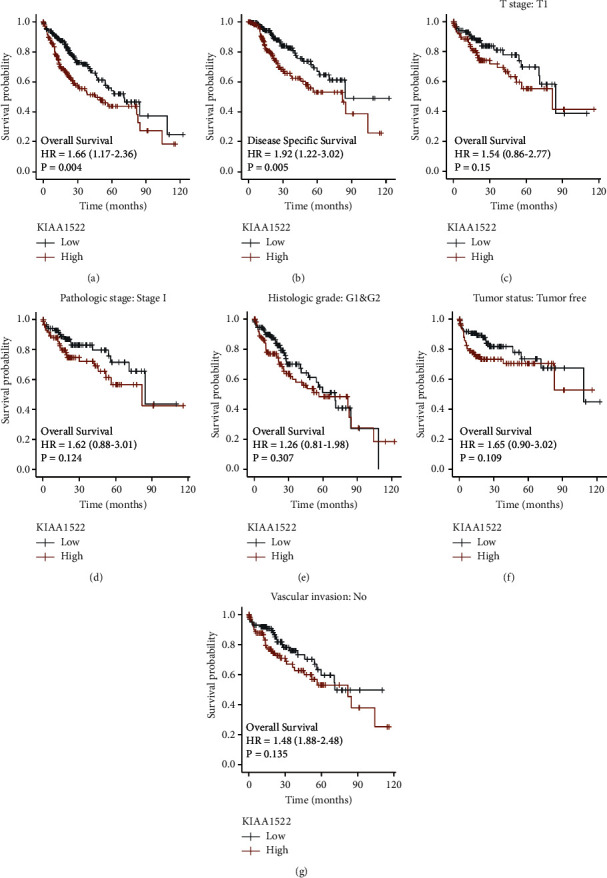
The prognostic value of KIAA1522 in different subgroups. (a, b) The prognostic value of KIAA1522 in OS and DSS of HCC. (c)–(g) The effects of KIAA1522 on OS in different subgroups.

The multivariate Cox regression includes every variable from the univariate Cox regression. T stage (*P*=0.027) and KIAA1522 (*P*=0.027) were found to be independent predictive variables for OS in multivariate Cox regression (Figure 3(a)). According to the survival status of HCC patients and the expression of KIAA1522, Figure 3(b) depicts the distribution of KIAA1522. The blue and orange dots represent HCC patients who are still alive and those who have passed away. The upper line shows median risk, while the left and right of the dashed line reflect low and high expressions of KIAA1522, respectively. Figure 3(b) shows that patients in the high-risk group had lower survival rates and a higher probability of passing away. By using diagnostic ROC analysis, the expression of KIAA1522 has some precision and accuracy for the diagnosis of HCC (Figure 3(c)). By using time-dependent ROC analysis, we also came to the conclusion that the expression of KIAA1522 showed some predictive power for HCC patients (Figure 3(d)). HCC patients were separated into two groups based on the median expression of KIAA1522, high expression and low expression. The high-expression group showed significant OS and disease-specific survival (DSS) correlations (Figures 4(a) and 4(b)).

### 3.3. Relationship between KIAA1522 Expression and Immune Infiltration

**Figure 5:**
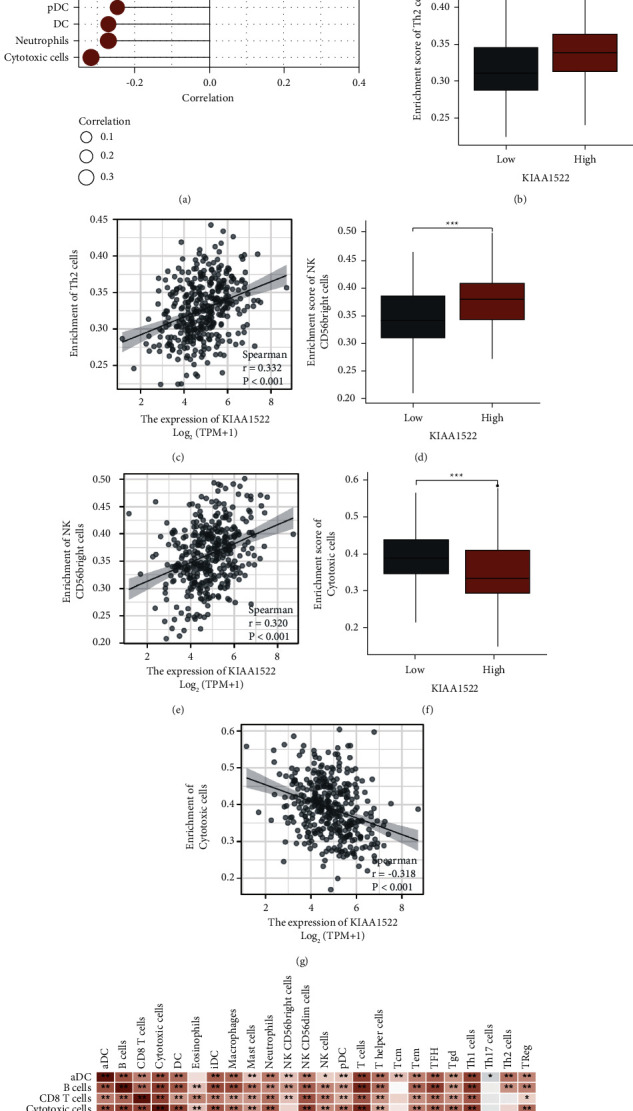
Results of the analysis between KIAA1522 expression and immune infiltration. (a) Correlation between KIAA1522 expression levels and relative abundance of immune cells. (b) Infiltration levels of Th2 cells in different KIAA1522 groups. (c) Positive correlation between KIAA1522 and Th2 cells. (d) Infiltration levels of NK CD58 bright cells in different KIAA1522 expression groups. (e) Positive correlation between KIAA1522 and NK CD58 bright cells in positive correlation. (f) Infiltration levels of cytotoxic cells in different KIAA1522 expression groups. (g) Negative correlation of KIAA1522 with cytotoxic cells. (h) Heat map of KIAA1522 expression levels in immune infiltrating cells in HCC.

We examined the relationship between KIAA1522 expression levels and the infiltration levels of 24 immune cells using the ssGSEA method (Figure 5(a)). In particular, the high KIAA1522 expression group had greater numbers of Th2 cells and NK CD58 bright cells infiltration (*P* < 0.001) (Figures 5(b) and 5(d)), and these findings demonstrated a strong positive link between these two factors (Figures 5(c) and 5(e)). In contrast, the level of cytotoxic cell infiltration was significantly lower (*P* < 0.001) in the group with high KIAA1522 expression (Figure 5(f)) and correlated negatively with KIAA1522 expression (Figure 5(g)). KIAA1522 was found favorably connected with the remaining immune cells, including T helper cells, follicular helper T (TFH) cells, activated dendritic cells (aDC), and macrophages. However, some immune cells, including neutrophils, NK cells, T helper 17 (Th17) cells, plasmacytoid DC (pDC), dendritic cells (DC), CD8 T cells, and T gamma delta (Tgd), were adversely linked with KIAA1522. The heat map evaluated the degree of connection between KIAA1522 ratios and 24 subgroups of immune cells that infiltrate tumors (Figure 5(h)). The findings revealed that KIAA1522 was crucial to HCC's immune infiltration.

### 3.4. Functional Enrichment Analysis of DEGs

**Figure 6:**
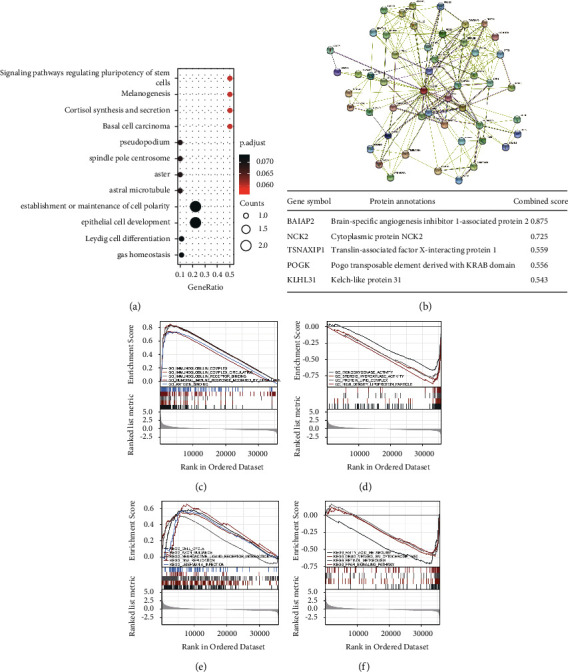
Enrichment analysis of KIAA1522 in HCC. (a) Biological process enrichment related to KIAA1522-related genes. (b) A network of KIAA1522 and its 20 potential cointeraction proteins. (c)–(f) The results of enrichment analysis from GSEA.

KIAA1522 may be implicated in signaling pathways that control basal cell carcinoma, melanogenesis, cortisol synthesis and secretion, stem cell pluripotency, and cortisol secretion, according to analysis through the Gene Ontology (GO) database (Figure 6(a)). Using the STRING database, we examined the function of KIAA1522 and its probable coexpressed genes in KIAA1522-related proteins (Figure 6(b)). The outcomes demonstrated that the top 5 subunit proteins interacting with KIAA1522 were BAIAP2, NCK2, TSNAXIP1, POGK, and KLHL31. Enrichment study of KIAA1522 connected with GO and KEGG pathways (given in Supplemental Table S1), which were positively associated, comprised 5 immune-related complexes and humoral immune responses mediated by circulating immunoglobulins and antigen binding (Figures 6(c) and 6(e)), while negatively correlated with monooxygenase activity, fatty acid metabolism, and PPAR signaling pathway (Figures 6(d) and 6(f)).

## 4. Discussion

The gene for the intercellular adhesion-mediatingligand-protein KIAA1522 is found in the region of chromosome 1P35.5 that spans the distances of 33.203 K to 33.245 kbp. The four transcripts T1, T2, T3, and T4 have been shown to encode peptides of 1094, 1046, 1035, and 143 amino acids, respectively, for four isomer proteins. By sequencing human tissues, KIAA1522 was discovered in 2000; however, its biological function is mostly unknown [27]. When Chen et al. examined the genome-wide methylation status in tissues from Kazakh patients with esophageal squamous cell carcinoma (ESCC) in Xinjiang, they discovered that the KIAA1522 gene's promoter region was heavily methylated [28]. The ERK signaling pathway was subsequently activated by KIAA1522 overexpression in ESCC tissues, which was shown to enhance ESCC cell differentiation, appreciation, and metastasis both in vivo and in vitro [13]. Additionally, Liu et al. looked at the expression of KIAA1522 in 583 paired tissues and discovered that it was overexpressed in tissues from nonsmall cell lung cancer (NSCLC) patients and that KIAA1522 expression was a prognostic indicator [29, 30]. Positive expression of KIAA1522 in colorectal cancer tissues was associated with distant metastasis of tumors and poor patient prognosis [31]; similarly, KIAA1522 is persistently increased in triple-negative breast cancer tissues, and its expression level may serve as a predictor of visceral metastasis in breast cancer [32]. According to the research, KIAA1522 promotes tumor growth in a number of malignancies.

In this investigation, we discovered that high expression of KIAA1522 in HCC patients was consistent with the level of conventional diagnostic marker and was linked with numerous advanced cancer clinical aspects (such as pathological stage, *T* stage histological grade, and poor OS). In order to account for the effects of age and gender on unfavorable outcomes, AFP in serum, univariate, and multivariate analyses were carried out, which suggests that KIAA1522 is a possible prognostic and diagnostic marker. Recent reports [14, 15] agree with the findings.

The development of HCC is controlled by the dynamic interaction between immune cells and cancerous cells in the immunological infiltrative tumor microenvironment [33]. However, there is no information on the connection between KIAA1522 and immune defense or invasion in HCC. The relationship between the expression of KIAA1522 and the degree of immune infiltration in HCC was examined using the ssGSEA. It was discovered that KIAA1522 had a mildly positive correlation with aDC and iDC and a substantial positive correlation with Th2 cells, NK CD56 bright cells, T helper cells, TFH, macrophages, and Th1 cells. According to certain reports, IL-4, IL-8, and IL-10 released by Th2 cells may have anti-inflammatory effects that aid in the development of HCC [34]. The immune system is suppressed by Th2 cells, which are helper T cells that cause M1 macrophages to polarize toward immunosuppressive M2 macrophages, supporting tumor genesis [35]. Therefore, we conclude that increased Th2 plays an aggressive role in the development of HCC as a result of the high expression of KIAA1522. Large numbers of NK cells that have infiltrated tumors have been found to have a regulatory role in tumor growth rather than aid in the fight against HCC, according to recent research. Specific subsets of NK cells that are substantially enriched in CD56 bright subsets may play a crucial role in the maintenance of endothelial proliferation, cancer cell migration, and HCC metastasis [36]. These data were useful in explaining the immune infiltration analysis's discovery of a high positive connection between KIAA1522 and NK CD56 bright cells.

Neutrophils, Tgd, DC, pDC, and cytotoxic cells all had negative correlations with KIAA1522. Tumor-infiltrating DCs are useful as a prognostic marker for HCC because they are inversely linked with tumor progression in most malignancies due to the crucial regulatory role of DCs bridging innate and adaptive immunity. Additionally, there was a strong correlation between the frequency of cytotoxic cells and the number of DC in tumors. Patients who had high levels of these two cells in their bodies typically had a more favorable prognosis for tumor-free survival [33]. The IFN-I pair produced by the pDC has positive anticancer action, and it is crucial to the immune system's ability to defend against cancer [37].

The finding that KIAA1522 is inversely linked with neutrophils is debatable. Neutrophil infiltration is currently thought to be a sign of a bad prognosis in HCC. Through the dual impacts of extracellular traps on cancer and endothelial cells, it can cause tumor metastasis [38]. Large quantities of bone morphogenetic protein 2 (BMP-2) and tumor necrosis factor *β*2 (TGF-*β*2) are secreted during the neutrophil invasion of the tumor microenvironment, which causes microRNA-301-3p expression in HCC cells. It gives HCC a stem-cell-like phenotype by suppressing the expression of cylindromatosis and limb-related membrane proteins in the tumor [39]. In order to encourage the growth and spread of tumors, it can also release angiogenic substances [40]. The impact of neutrophils on malignancies, however, is a subject of debate [41, 42]. Neutrophil invasion can promote tumor cell apoptosis by ROS damage [43] and positively influence adaptive immunity, including tumor immunity, by interacting directly with lymphocytes and DCs and indirectly by generating lymphocytes/DCs-related bioactive molecules [44]. Depletion of neutrophils prevents the development and effectiveness of immunization against tumor antigens [45]. Neutrophils can take up exogenous antigens in vivo, present them to MHC I molecules to activate naive T cells [46], and transmit the antigens to memory CD4+ lymphocytes in an MHC class II-dependent manner [47]. In vitro, GM-CSF can cause neutrophils to differentiate into heterozygotes that have both neutrophil and DC features, improving the immune cells' capacity for antigen presentation [48]. Chemokine receptor 7 (CCR7)-induced neutrophil migration to draining lymph nodes improves the ability of neutrophils to deliver antigen and encourages the development of tumor immunity [49]. Therefore, more research needs to be done to determine how the interaction between KIAA1522 and neutrophils affects the growth of HCC. KIAA1522, however, was discovered to be enriched in a number of immune complex formation-related pathways, indicating that it may have played a role in the immune response during the growth of HCC.

HCC formation and recurrence is a complicated, multilayered process influenced by numerous variables [50]. According to the formation of the PPI network, KIAA1522 primarily interacts with NCK2 and BAIAP2 (combined score >0.7). In cancer research, two proteins have been identified as having potential tumor-promoting properties. The major risk factor for a bad outcome in colorectal cancer is abnormal localization of BAIAP2 on the cell membrane [51]. The intestinal microbiome characteristics of hepatic fibrosis patients that enhance oxidative stress and an inflammatory environment are related to the NCK2 single nucleotide polymorphisms RS3769502 and RS7573751 [52]. To create endothelium podosomes in vitro, NCK2 also engages in an interaction with Dok1, a downstream tyrosine kinase 1. The extracellular matrix (ECM) is broken down by the actin remodeling process to create podosomes, a membrane-bound adhesion structure that facilitates cancer cell invasion [53]. Additionally, NCK2 can be a significant contributor in enhancing tumor survival and aggressiveness in ovarian cancer [54].

The GSEA study of the underlying mechanism of KIAA1522 revealed that KIAA1522 was negatively linked with fatty acid metabolism and PPAR pathway activation and favorably enriched in cancer development-related pathways, such as cell cycle and DNA replication. Fatty acids are essential for the development of cell membranes, the storage of energy, and the synthesis of signal molecules in cancer cells. As a result, cancer cells have the potential to damage and proliferate at several sites in the fatty acid metabolic pathway, and limiting the availability of fatty acids can effectively stop the spread of cancer cells [55]. Given that fatty acid metabolism and KIAA1522 have a negative association, it is possible to decrease fatty acid breakdown to achieve KIAA1522's proliferative effect on HCC cells. The PPAR signaling pathway is essential for defending the liver against pathological assaults such as inflammation and cancer injuries [56], and KIAA1522's suppression of this pathway may increase the chance of unfavorable outcomes in HCC patients.

Overall, our research highlights the critical function of KIAA1522 in HCC. Increased KIAA1522 expression was linked to worse prognostic outcomes in HCC patients, and a relationship between KIAA1522 and immune cells that invade tumors was discovered. Understanding the various methods by which KIAA1522 impacts HCC will be aided by the discovery made by the underlying mechanism analysis that the compound is engaged in tumor-related pathways such as DNA replication, cell cycle, immune response, PPAR signaling pathway, and suppression of fatty acid metabolism. This study has some limitations, including the fact that the results are primarily drawn from a public database of pathological tissues, whether KIAA1522 in blood also has equivalent diagnostic and prognostic value, and the need for additional in vitro and in vivo experiments to confirm the mechanistic analysis is used in this study.

## 5. Conclusions

High levels of KIAA1522 expression could contribute to tumorigenesis, be linked to innate immunity, such as immune cell infiltration and humoral immunity, and have an impact on HCC by inhibiting tumor suppressor-related pathways like fatty acid metabolism and liver protective mechanisms like the PPAR signaling pathway.

## Figures and Tables

**Table 1:** Demographic and clinicopathological parameters of high and low KIAA1522 expression group patients with hepatocellular carcinoma in TCGA-LIHC.

Characteristic	Low expression of KIAA1522	High expression of KIAA1522	*P* value
*N*	187	187	
T stage, *n* (%)			0.031
T1	105 (28.3%)	78 (21%)	
T2	40 (10.8%)	55 (14.8%)	
T3	33 (8.9%)	47 (12.7%)	
T4	6 (1.6%)	7 (1.9%)	
N stage, *n* (%)			1.000
N0	119 (46.1%)	135 (52.3%)	
N1	2 (0.8%)	2 (0.8%)	
M stage, *n* (%)			1.000
M0	125 (46%)	143 (52.6%)	
M1	2 (0.7%)	2 (0.7%)	
Pathologic stage, *n* (%)			0.070
Stage I	98 (28%)	75 (21.4%)	
Stage II	39 (11.1%)	48 (13.7%)	
Stage III	35 (10%)	50 (14.3%)	
Stage IV	3 (0.9%)	2 (0.6%)	
Gender, *n* (%)			0.047
Female	51 (13.6%)	70 (18.7%)	
Male	136 (36.4%)	117 (31.3%)	
OS event, *n* (%)			0.039
Alive	132 (35.3%)	112 (29.9%)	
Dead	55 (14.7%)	75 (20.1%)	
Vascular invasion, *n* (%)			0.104
No	114 (35.8%)	94 (29.6%)	
Yes	49 (15.4%)	61 (19.2%)	
Histologic grade, *n* (%)			<0.001
G1	41 (11.1%)	14 (3.8%)	
G2	100 (27.1%)	78 (21.1%)	
G3	39 (10.6%)	85 (23%)	
G4	4 (1.1%)	8 (2.2%)	
Age, median (IQR)	64 (53, 70)	59 (51, 68)	0.020
AFP(ng/ml), median (IQR)	7 (3.5, 37.5)	47 (7, 1902)	<0.001
BMI, median (IQR)	25.71 (22.92, 30.1)	23.42 (20.79, 27.32)	<0.001

**Table 2:** The relationship between KIAA1522 expression and clinicopathological features.

Characteristics	Total (*N*)	Odds ratio (OR)	*P* value
T stage (T2 and T3 and T4 vs. T1)	371	1.857 (1.232–2.811)	0.003
N stage (N1 vs. N0)	258	0.881 (0.104–7.439)	0.900
M stage (M1 vs. M0)	272	0.874 (0.104–7.372)	0.894
Pathologic stage (stage III and stage IV vs. stage I and stage II)	350	1.524 (0.942–2.484)	0.088
Histologic grade (G3 and G4 vs. G1 and G2)	369	3.315 (2.132–5.217)	<0.001
Vascular invasion (yes vs. no)	318	1.510 (0.950–2.410)	0.082
AFP (ng/ml) (>400 vs.≤400)	280	3.997 (2.196–7.577)	<0.001
Albumin (g/dl) (≥3.5 vs. <3.5)	300	1.354 (0.788–2.354)	0.276
Tumor status (with tumor vs. tumor free)	355	1.640 (1.076–2.511)	0.022

**Table 3:** Univariate analysis of HCC patients.

Characteristics	Total (*N*)	Hazard ratio (95% CI)	*P* value
Age	373		
≤60	177	Reference	
>60	196	1.205 (0.850–1.708)	0.295
Gender	373		
Female	121	Reference	
Male	252	0.793 (0.557–1.130)	0.200
Histologic grade	368		
G1	55	Reference	
G2	178	1.162 (0.686–1.968)	0.577
G3&G4	135	1.222 (0.710–2.103)	0.469
T stage	370		
T1 and T2	277	Reference	
T3 and T4	93	2.598 (1.826–3.697)	**<0.001**
M stage	272		
M0	268	Reference	
M1	4	4.077 (1.281–12.973)	**0.017**
N stage	258		
N0	254	Reference	
N1	4	2.029 (0.497–8.281)	0.324
KIAA1522	373	1.343 (1.136–1.587)	**<0.001**
Pathologic stage	349		
Stage I	173	Reference	
Stage III and stage II and stage IV	176	2.090 (1.429–3.055)	**<0.001**

The bold fonts were used to highlight the factors influencing the survival of HCC patients in univariate Cox regression,

## Data Availability

The datasets used and analyzed during the current study are available from the corresponding authors upon request.

## Supplementary Materials

Supplementary Materials Table S1. Details of signaling pathways significantly associated with KIAA1522 expression.
