# Supplementary material for: Poor Prognostic Biomarker KIAA1522 Is Associated with Immune Infiltrates in Hepatocellular Carcinoma
Source: J Oncol. 2023 Jan 31;2023:3538928. doi: 10.1155/2023/3538928 (PMC9904920; doi:10.1155/2023/3538928)
Supplement: Supplementary Materials — Table S1. Details of signaling pathways significantly associated with KIAA1522 expression. [file 3538928.f1.pdf]

Table S1. Signaling pathways are significantly associated with KIAA1522 expression.

Description	SetSize	NES	p.adjust	rank	leading_edge
GO_IMMUNOGLOBULIN_COMPLEX_CIRCULATING	86	1.631448633	0.035018887	3447	tags=28%, list=10%, signal=25%
GO_IMMUNOGLOBULIN_COMPLEX	86	1.631451507	0.035018887	13593	tags=66%, list=38%, signal=41%
GO_IMMUNOGLOBULIN_RECEPTOR_BINDING	58	1.630511383	0.035149891	5358	tags=26%, list=15%, signal=22%
GO_HUMORAL_IMMUNE_RESPONSE_MEDIATED_BY_CIRCULATING_IMMUNOGLOBULIN	15	1.634529021	0.03481432	2368	tags=33%, list=7%, signal=31%
GO_ANTIGEN_BINDING	276	2.125666346	0.020126438	8480	tags=41%, list=24%, signal=31%
GO_MONOOXYGENASE_ACTIVITY	19	1.537748219	0.055497402	7047	tags=47%, list=20%, signal=38%
GO_EYE_PHOTORECEPTOR_CELL_DIFFERENTIATION	38	1.706983826	0.026827951	1683	tags=66%, list=5%, signal=63%
GO_EXTRACELLULAR_TRANSPORT	39	1.708317735	0.026827951	2562	tags=56%, list=7%, signal=52%
GO_HIGH_DENSITY_LIPOPROTEIN_PARTICLE	13	1.646896642	0.032895702	3483	tags=38%, list=10%, signal=35%
KEGG_DNA_REPLICATION	36	2.023524517	0.012754269	7556	tags=64%, list=21%, signal=50%
KEGG_LEISHMANIA_INFECTION	70	2.04761528	0.012754269	6176	tags=49%, list=17%, signal=40%
KEGG_NEUROACTIVE_LIGAND_RECEPTOR_INTERACTION	270	2.141688246	0.012754269	4629	tags=34%, list=13%, signal=30%
KEGG_AXON_GUIDANCE	129	2.146674673	0.012754269	5704	tags=29%, list=16%, signal=25%
KEGG_CELL_CYCLE	124	2.245176909	0.012754269	7999	tags=50%, list=22%, signal=39%
KEGG_FATTY_ACID_METABOLISM	42	-2.743236611	0.020809665	3078	tags=60%, list=9%, signal=54%
KEGG_DRUG_METABOLISM_CYTOCHROME_P450	71	-2.574891141	0.023174647	1462	tags=46%, list=4%, signal=45%
KEGG_RETINOL_METABOLISM	64	-2.458856538	0.023089127	1696	tags=45%, list=5%, signal=43%
KEGG_PPAR_SIGNALING_PATHWAY	69	-2.392889474	0.023089127	2577	tags=42%, list=7%, signal=39%
